# Neurotrophin Genes and Antidepressant-Worsening Suicidal Ideation: A Prospective Case-Control Study

**DOI:** 10.1093/ijnp/pyw059

**Published:** 2016-07-04

**Authors:** Géraldine Voegeli, Nicolas Ramoz, Tatyana Shekhtman, Philippe Courtet, Philip Gorwood, John R. Kelsoe

**Affiliations:** Department of Psychiatry, VA San Diego Healthcare System, La Jolla, CA (Drs Voegeli and Kelsoe); Department of Psychiatry (Dr Kelsoe), and Institute for Genomic Medicine (Ms Shekhtman and Dr Kelsoe), University of California San Diego, La Jolla, CA; INSERM U675, Center for Psychiatry and Neuroscience, Sainte-Anne Hospital, Paris, France (Drs Voegeli, Ramoz, and Gorwood); CMME, Sainte-Anne Hospital, Paris, France (Drs Voegeli and Gorwood); Department of Emergency Psychiatry and Acute Care, CHRU Montpellier, Inserm U1061, University of Montpellier, Montpellier, France (Dr Courtet).

**Keywords:** suicide, depression, antidepressant drugs, pharmacogenetics, neutrotrophin

## Abstract

**Background:**

Antidepressant-worsening suicidal ideation is a rare but serious phenomenon. This study aimed to test for association between antidepressant-worsening suicidal ideation and polymorphisms of BDNF/NTRK2 neurotrophin pathway genes, known to be involved in depression and suicide.

**Methods:**

This was a case-control study comparing patients with antidepressant-worsening suicidal ideation to patients without. Patients were collected from the GENESE cohort (3771 depressed tianeptine-treated outpatients). Antidepressant-worsening suicidal ideation was defined by an increase of at least 2 points on the Montgomery-Åsberg Depression Rating Scale-item10 during treatment. Controls were matched for age, sex, and baseline Montgomery-Åsberg Depression Rating Scale-item10 score. Thirteen single nucleotide polymorphisms covering 5 BDNF/NTRK2 pathway genes were genotyped.

**Results:**

A total 78 cases and 312 controls were included. Two NTRK2 single nucleotide polymorphisms were associated to antidepressant-worsening suicidal ideation: rs1439050 (*P=.*01) and rs1867283 (*P=.*04). Association with rs1439050 remained significant after adjustment for potentially confounding factors, including previous suicide attempts (*P<.*01).

**Conclusions:**

This naturalistic prospective study is consistent with previous studies on highlighting the potential role of the neurotrophin pathway, and especially of NTRK2, in antidepressant-worsening suicidal ideation.

## Introduction

Increase of suicidal thoughts during antidepressant treatment is a rare but serious phenomenon that has been reported for various antidepressant drugs such as selective serotonin reuptake inhibitors (Zisook et al., 2009) but also tricyclic antidepressants (Perroud et al., 2009), serotonin and norepinephrine reuptake inhibitors (Baek et al., 2015), or various antidepressants (Courtet et al., 2014), and which has led the FDA to issue a black box warning in 2006. Genetic studies, as adoption (Petersen et al., 2013) or twin studies (Voracek and Loibl, 2007), have shown the role of genetics in suicidal behavior, independently from the environment and from mood disorders (Brent and Mann, 2006). Among the different pathways involved in depression and suicidal behavior, the neurotrophin pathway, also known as BDNF/NTRK2 pathway, is a major one to consider. Indeed, in depression, both neurotrophic factor BDNF and its high-affinity receptor TrkB have been shown to be decreased, at both the RNA and protein level (Tripp et al., 2012). This decrease is reversible with antidepressant treatment (Huang et al., 2008). Siuciak et al. (1997) even showed an antidepressant-like effect of a brain BDNF-infusion in rodents. Molecular mechanisms of suicide also involve the BDNF-NTRK2 pathway. A decrease of BDNF and NTRK2 transcription levels (Dwivedi et al., 2009), as well as qualitative impairments such as decrease of autophosphorylation capacity of TrkB, have been found in postmortem suicide brains (Pandey et al., 2008).

Recent pharmacogenetic studies suggest that antidepressant-worsening suicidal ideation (AWSI) could be genetically driven (Laje et al., 2007; Brent et al., 2010). Some of these studies involved genes of the neurotrophin pathway, such as BDNF, NTRK2, or transcription factor CREB. Even if AWSI was initially described in adolescents and young adults under 25 years, this phenomenon could also occur in adults above 25 years, as few studies specifically investigated the risk of AWSI in adult populations. Apart from the large STAR*D (Perlis RH et al., 2007) and GENDEP (Perroud et al., 2009a) consortia, reporting up to 20% patients experiencing AWSI, the limited number of large prospective studies probably reduced the chances to replicate or invalidate such results in older subjects. Therefore, in a large naturalistic cohort of depressed outpatients treated by tianeptine, we tested the association between common variants of genes of the neurotrophin pathway and the occurrence of AWSI.

## Methods

### Sample

GENESE is a large, prospective, naturalistic cohort of 3771 French outpatients diagnosed with major depression and treated with tianeptine, one of the most prescribed antidepressants among French general practitioners (GPs) (Hérique and Kahn, 2009). Dosage of tianeptine was managed by a GP and had to range between 12.5 and 37.5mg/d, according to prescription recommendations. During the first visit, GPs validated the diagnosis of major depressive episode (MDE) by checking each DSM-IV criteria for MDE and checked the other inclusion criteria (age above 18 years and Caucasian ethnicity) and the absence of DSM-IV alcohol dependence. They rated suicidal thoughts using a single item (item 10, “suicidal thoughts”) of the Montgomery-Åsberg Depression Rating Scale (MADRS). Patients self-evaluated depressive symptoms at baseline using the Hospital Anxiety and Depression Scale, then filled in item 10 of the MADRS at day 14. Hospital Anxiety and Depression Scale and MADRS item 10 were finally rated by GPs during the last visit (between day 42 and day 56) to assess the evolution of the MDE. DNA was collected at baseline by buccal swab.

AWSI was defined as an increase of at least 2 points on the MADRS-item10 between baseline and day 14 or final visit. The other inclusion criteria were age above 18 years and Caucasian ethnicity. We did not distinguish treatment “emergent” suicidal ideation from treatment “worsening” suicidal ideation, as Perroud et al. (2009b) showed that both notions were not referring to distinct phenotypes. We selected controls matched for age, sex, and severity of suicidal thoughts on MADRS-item 10 at baseline. A ratio of 4 controls for 1 case was chosen to increase statistical power.

### Single Nucleotide Polymorphisms (SNP) Selection

SNPs selection was made according to 4 criteria: (1) SNPs of neurotrophin pathway genes that had been previously reported as associated with either suicide or AWSI, (2) minor allele frequency >5%, (3) distance between 2 SNPs >10 000bp to limit linkage disequilibrium, and (4) total number of SNPs <20 because of the moderate size of our sample of cases. The priority was given to NTRK2 while choosing SNPs, considering its major role in antidepressant molecular mechanism of action. A total of 13 SNPs was therefore included: rs6265 and rs962369 from *BDNF*; rs10868235, rs1114800, rs1867283, rs1147198, rs1187286, rs1439050, and rs1387923 from NTRK2; rs2072446 from *p75NTR* (BDNF and NGF low affinity receptor); rs2551919 and rs4675690 from *CREB* (transcription factor downstream from BDNF); and rs1360550 from *PKC* (involved in NTRK2 PLCγ signal cascade).

### Genotyping

Genotyping was performed using a 5’ exonuclease assay (Taqman, Life Technologies). Assay products were run on an Applied Biosystem 7900HT Fast Real-Time PCR System (Life Technologies).

### Statistics

The case-control single-SNP analysis was performed using a logistic regression model as implemented in PLINK (Purcell et al., 2007) (http://pngu.mgh.harvard.edu/~purcell/plink/). Empirical *P* values were estimated by permutation to lower the risk of chance finding due to multiple comparisons. Haplotypes analysis was also conducted using PLINK. The only results taken into account were the results of the omnibus test, which tests all observed haplotypes. Clinical data analyses used Student *t* test and chi-square test, and potentially confounding factors were controlled for by a logistic regression analysis.

### Ethics Statement

The study was performed according to French regulatory guidelines and current codes of Good Clinical Practice. Each patient was informed about the aims and procedures of the study and provided written, signed consent. The study protocol was submitted to and approved by local independent ethics committees (C.P.P., reference no. 08042.)

## Results

### Clinical Features

Seventy-eight patients met the criteria for the AWSI group and 312 patients were selected as controls, for a ratio of 4 controls/case. Mean age at inclusion was 48.2 years, and the sex ratio was 2 women for 1 man. Table 1 presents the socio-demographic and clinical features for both groups. Concerning current episode, alcohol abuse, and benzodiazepine coprescription were more often reported in cases than controls (5.3% vs 1.0%, respectively; *P*=.03 and 62.2% vs 40.8%, *P*<.01). Concerning mood disorder, the current episode was less frequently a first episode in cases (40.3% vs 57.4%, *P<.*01). Comparing the presence of all DSM-IV criteria of major depressive disorder, one item was more frequently rated for cases at baseline, namely agitation (85.3% vs 75.5%, *P*=.07), but the difference was not statistically significant. Lastly, a history of suicide attempt was much more frequent in cases than controls (26.0 vs 6.3, *P<.*01).

**Table 1.** Clinical Features of Both Groups

	AWSI Group	Control Group	*P* value	
	n=78	n=312		
Demographic characteristics				
Mean (SD)				
Current age (y)	50.0 (14.7)	47.7 (14.2)	.23	
n (%)				
Men	26 (33.3)	101 (32.4)	.87	
Current episode characteristics				
Mean (SD)				
Total baseline HAD score	26.3 (7.2)	24,9 (6.2)	.16	
Baseline depression HAD score	12.6 (4.4)	12.0 (3.9)	.30	
Baseline anxiety HAD score	13.7 (4.2)	12.9 (3.5)	.14	
Tianeptine dose (mg/d)	36.25 (7.1)	35(7.1)	.54	
n (%)				
Benzodiazepines coprescription	46 (62.2)	125 (40,8)	<.01	*
Alcohol abuse	4 (5.3)	3 (1.0)	.03	*
Mood disorder characteristics				
Mean (SD)				
Age at first episode	38.6 (14.9)	34.5 (14.2)	.16	
Total number of episodes	3.1 (3.6)	2.4 (1.6)	.17	
Cumulative duration of episodes (mo)	41.2 (34.3)	32.9 (26.3)	.18	
n (%)				
First episodes	31 (40.3)	178 (57.4)	<.01	*
Suicide attempts history	20 (26.0)	17 (5.6)	<.01	*

*P value < .05.

### Single SNP Association Analysis

Two SNPs were significantly associated with AWSI, both being located in the *NTRK2* gene (Table 2): rs1439050 (*P=.*01, 1/OR= 1.69) and rs1867283 (*P=.*04, 1/OR= 1.46). Only rs1439050 remained significant after the permutation process (*P=.*02). Given that only one SNP among all SNPs tested was a functional one, BDNF SNP rs6265, also known as Val66Met and involved in impaired processing of proBDNF and secretion of the processed peptide, we conducted a genotypic analysis for this particular SNP, which did not show any difference between Met/+ carriers and Val/Val homozygotes patients (*P*= .43, 1/OR = 1.36).

**Table 2.** Single SNP Analysis of 13 SNPs (Allelic Analysis)

Chromosome	Gene	SNP	Position (bp)	Allele	AWSI n (%)	Controls n (%)	*P* value	*P* value after Permutation	1/OR
2	CREB	rs2551919	208430383	C	128 (82.0)	518 (83.0)	*0.921*		0.98
				T	26 (17.3)	106 (17.0)			
		rs4675690	208507807	C	87(55.5)	334(53.6)	*0.676*		1.08
				T	69 (44.5)	290 (46.4)			
9	NTRK2	rs1147198	87275598	A	120 (76.7)	466 (74.7)	*0.627*		1.11
				C	36 (23.3)	158 (25.3)			
		rs1439050	87288193	**G**	**116 (74.67**)	**397 (63.6**)	0.010*	0.017*	**1.69**
				**T**	**40 (25.3**)	**227 (36.4**)			
		rs1187286	87415028	A	125 (80.0)	461 (74.0)	*0.124*		1.41
				C	31(20.0)	163 (26.1)			
		rs1867283	87450766	**A**	**62 (40.0**)	**308 (49.4**)	0.040*	*0.072*	**1.46**
				**G**	**94 (60.0**)	**316 (50.7**)			
		rs10868235	87493755	C	62 (40.0)	294 (47.1)	*0,119*		1.33
				T	94 (60.0)	330 (52.9)			
		rs11140800	87508137	A	94 (60.0)	380 (60.9)	*0.844*		0.96
				C	62 (40.0)	244 (39.1)			
		rs1387923	87640886	C	70 (44.7)	271 (43.4)	*0.773*		0.95
				T	86 (55.3)	353 (56.6)			
11	BDNF	rs6265	27679916	A	32 (20.7)	151 (24.2)	*0.361*		1.23
				G	124 (79.3)	473 (75.8)			
		rs962369	27734420	A	114 (73.3)	493 (79.1)	*0.129*		0.73
				G	42 (26.7)	131 (20.9)			
10	PKCε	rs1360550	30487760	C	64 (40.8)	250 (40.1)	*0.876*		0.97
				T	92 (59.2)	374 (59.9)			
17	p75NTR	rs2072446	49510457	C	146 (93.3)	583 (93.4)	*0.961*		0.98
				T	10 (6.7)	41 (6.6)			

*P value < .05.

### Adjustment for Potential Confounding Variables

Given the significant differences of some potentially contaminant clinical features between cases and controls, we conducted a covariate analysis. After adjustment for past suicide attempts, being in a first vs later depressive episode, presence of alcohol abuse, or benzodiazepine coprescription, rs1439050 was the only SNP remaining significant (*P<.*01, *P=.*02, *P=.*02, and *P=.*04, respectively). On the other hand, after adjustment on the “agitation” item at baseline, rs1439050 and rs1867283 remained associated to AWSI (*P=.*01 and *P*= .04, respectively).

### Subpopulation Analysis

Assessing the role of the rs1439050 G allele in different subpopulations (emergent vs worsening suicidal ideations, with vs without suicide attempts history, and younger vs older than 25 years old), we found that the association with rs1439050 was maintained for (1) patients experiencing worsening suicidal ideation during treatment, (2) patients older than 25 years, and (3) patients without a suicide attempt history (*P*=.02, OR=1.72; *P*=.01, OR=1.72; *P*=.01, OR = 1.72, all *P* values resisting permutation processes, respectively). Patients with emergent suicidal ideas, younger than 25 years, or with a suicide attempt history (with a respective number of cases of 22, 5, and 9) did not reach statistical significance (*P*=.18, OR=.62; *P*=.67, OR=.75; and *P*=.45, OR= .67, respectively).

### Haplotype Analysis

Three haplotypes, all composed of *NTRK2* markers, were associated with AWSI: only one involves rs1439050 (rs1439050|r1187286), with an omnibus test *P* =.03) lower than rs1439050 alone, in accordance with the partial linkage disequilibrium between these 2 SNPs. The other 2 haplotypes consisted of 2 or 3 NTRK2 SNPs (rs10868235|rs11140800, omnibus test *P* =.04, and rs1867283|rs10868235|rs1114080, omnibus test *P* =.02).

## Discussion

In this study, we replicate the findings of Perroud et al. (2009a) about *NTRK2* SNP rs1439050, as this particular polymorphism was associated with an increase of suicidal thoughts during the first weeks following the introduction of an antidepressant treatment in the present study. The associations in these 2 studies surprisingly go in a different direction (OR=0.587 in our study, OR=1.268 in the Perroud et al. study (2009b)), although both GENDEP and GENESE cohorts consist of Caucasian European patients and therefore have the same ethnicity basis. A potential explanation is that rs1439050, an intronic SNP with no specific reported role, is not a vulnerability factor per se (i.e., directly explaining the phenotype) but rather is in linkage disequilibrium with a nearby unidentified functional mutation of NTRK2, tagged by the same marker, although with a different allele. The nearest SNPs known to induce translation errors (missenses) are rs748182988 and rs201875843, with respective positions on chromosome 9 84670950 and 84670940, while the rs1439050 position on chromosome 9 is 84673278.

Interestingly, *NTRK2* seems to be involved in AWSI regardless of the class of antidepressant, as the association has been reported for a serotonin reuptake inhibitor (escitalopram), a tricyclic antidepressant (nortriptyline) in the study of Perroud et al. (2009a), and now tianeptine, an atypical antidepressant whose chemical structure is close to tricyclics. The actual molecular mechanism of action of antidepressants on the neurotrophin pathway remains unclear, but some studies have recently suggested that TrkB could play a major role, independently of BDNF binding. Rantamäki et al. (2011) showed that administration of antidepressants to rodents induced an increase of the phosphorylation level of TrkB, while BDNF transcription levels remained unchanged. This phenomenon was even found to occur in BDNF knockout mice, confirming that BDNF binding is not necessary for short-term TrkB activation by antidepressants. Even if the actual mechanism of autophosphorylation of TrkB by antidepressants remains unsolved, as no binding of antidepressants on TrkB has ever been found, this activation was interestingly replicated for several classes of antidepressants (Rantamäki et al., 2007). A functional alteration of TrkB could then affect the action of antidepressants on the neurotrophin pathway and participate in the emergence of side effects such as AWSI.

Interestingly, although having a suicide attempt history is known to be a major risk-factor for suicide and AWSI (Courtet et al., 2014), association between rs1439050 and AWSI resisted adjustment for this confounding variable. Adjustment for alcohol abuse or benzodiazepine prescription, both higher in cases, did not alter this association either. The analyses on different subpopulations showed that the association with rs1439050 was maintained for patients (1) having experienced worsening suicidal ideation, (2) older than 25 years old, and (3) without suicide attempts history, but did not reach statistical significance for patients (1) with emergent suicidal ideation, (2) younger than 25 years, and (3) with a suicide attempt history. However, the small number of cases in each of these latter subgroups (22, 5, and 9 patients, respectively) might partly explain this result because of a lack of statistical power. Overall, our clinical features did not determine a clear profile of patients experiencing AWSI. While the trend toward a higher rate of agitation at diagnosis among cases could support the hypothesis of mixed states, mistaken for MDEs and wrongly treated by antidepressants (Akiskal et al., 2005), the lower rate of first episodes in cases as well as the trend toward a higher number of depressive episodes in their history and a longer cumulative duration of depressive episodes contradict this argument and defend the hypothesis of severe multi-treated patients, who might have developed lower sensitivity to antidepressants. Besides, this last hypothesis is reinforced by the fact that adjustment on the “agitation” item does not alter the statistical association between AWSI and the rs1439050 SNP. The worsening of suicidal thoughts could then be explained by the lack of efficacy of antidepressant drugs on an evolving depressive episode, as supposed by Courtet et al. (2014).

There are several limitations to this study. The main one is the modest size of our cases sample, despite the very large cohort sample from which they were selected, due to the rarity of this phenomenon. However, the prospective and naturalistic design of this study, and therefore the good reliability and representativeness of the data, partly balance this lack of statistical power.

Second, our AWSI prevalence (1.8% of the cohort) is lower than those reported in the literature (4–20%) (Perroud et al., 2009b). One explanation is that GENESE is an outpatient cohort, which excludes the most severe depressions, perhaps more exposed to AWSI. Another explanation could be a lack of detection of this phenomenon: indeed, rating of suicidal thoughts was performed by GPs at baseline and final visit or by patients themselves at day 14. Both are less accustomed to MADRS rating than specialists, which could have induced a detection bias. Nevertheless, studies have shown that a single item rating was as valid approach to assess suicidal thoughts (Desseilles et al., 2012) and that self-assessment could be as accurate as hetero-evaluation (Moroge et al., 2014). Moreover, MADRS scale was first intended to be assessed by trained as well as less-trained caregivers (Davidson et al., 1986). Nevertheless, intra-rater reliability might be more important in the present study, as the definition of the AWSI phenotype is based on a rating difference between two different time points.

Third, no consensus exists about the definition of AWSI. In this study, we chose to favor specificity in the definition of the AWSI phenotype at the risk of reducing sensitivity. A larger definition of AWSI would have raised our prevalence of AWSI: taking an increase of at least 1 point instead of 2 on MADRS-item10 as AWSI definition would have risen our prevalence to 4.0%, which is more consistent with the literature but which also potentially implies decreased specificity.

In conclusion, this study reports an association between AWSI and SNP rs1439050 of *NTRK2* in depressed subjects treated by tianeptine. Our findings support the hypothesis of a major role of *TrkB* in antidepressant drugs mechanism of action and therefore in AWSI, independently of its activation by BDNF binding, and whatever the class of antidepressant considered.

## Statement of Interest

Geraldine Voegeli, John Kelsoe, N. Ramoz, and Tatyana Shekhtman: none. Philip Gorwood received research grants from Eli Lilly and Servier; honoraria for presentations in congresses from AstraZeneca, Bristol-Myers Squibb, Janssen, Lundbeck, and Servier; participated in advisory board with AstraZeneca, Janssen, Roche, and Servier; and has a paid position at University of Paris-Descartes and hospital Sainte-Anne, and no shares. Philippe Courtet received research grants from Eli Lilly and Servier; honoraria for presentations in congresses from AstraZeneca, Bristol-Myers Squibb, Lundbeck, and Servier; and has a paid position at University of Montpellier and CHU Montpellier, and no shares.
